# Oxide Removal Mechanism and Process Optimization During Integrated Pulsed-Continuous Laser Cleaning of Q235B Carbon Steel

**DOI:** 10.3390/ma18061247

**Published:** 2025-03-12

**Authors:** Wei Zhang, Chunming Wang, Qiong Wu, Fei Yan, Guoli Zhu, Junqiang Wang

**Affiliations:** 1 School of Mechanical Science and Engineering, Huazhong University of Science and Technology, Wuhan 430074, China; zhangweid2022@hust.edu.cn (W.Z.); glzhu@mail.hust.edu.cn (G.Z.); 2 School of Materials Science and Engineering, Huazhong University of Science and Technology, Wuhan 430074, China; cmwang@hust.edu.cn; 3 School of Automotive Engineering, Wuhan University of Technology, Wuhan 430070, China; wuqiong19556450725@163.com; 4 School of Physics and Electronic Information, Luoyang Normal University, Luoyang 471934, China; wjq@lynu.edu.cn

**Keywords:** integrated pulsed-continuous laser cleaning, Q235B carbon steel, surface quality, microstructure, oxide removal mechanism

## Abstract

Laser cleaning has received extensive attention due to its high efficiency, non-pollution and easy automation. However, how to improve the cleaning quality has become the focus of current research. In this paper, we used a pulsed laser for cleaning experiments on Q235B carbon steel to investigate the effects of different process parameters on the surface cleaning quality. On this basis, a new cleaning method was innovatively proposed to improve the oxide removal efficiency, microstructure, and mechanical properties of cleaned samples. The results showed that pulsed laser cleaning of Q235B carbon steel was the most effective at a laser linewidth of 50 mm, pulsed frequency of 500 kHz, and cleaning speed of 15 mm/s. A great deal of craters formed on the surface of cleaned samples due to the thermal shock of the pulsed laser. Compared with other laser cleaning methods, integrated laser cleaning had an obvious effect in raising the oxide removal efficiency and reducing the surface roughness. The ridge structures on the sample surface also could be successfully eliminated, subsequently achieving smooth structures. Fine-crystalline structures were formed near the surface of tested samples, which significantly decreased the crystal orientation and increased the number of small angle grain boundaries and the GND density. The improvement in hardness was mainly on account of grain refinement in the integrated laser cleaning samples. In addition, a physical model was proposed to illustrate the oxide removal mechanism on integrated pulsed-continuous laser cleaning samples. This research can offer new theoretical and technical support for solving the long-standing problems of efficiency and quality in laser cleaning, thus significantly broadening the application of laser technology in manufacturing fields.

## 1. Introduction

In recent years, Q235B carbon steel has attracted widespread attention on account of its excellent strength, toughness, and extensibility, and it is extensively used for fabricating machine parts, pressure equipment, aerospace structures, and other parts [1,2]. It is prone to oxidation when frequently exposed to the air or hot rolling at high temperature, thus leading to a marked decrease in its service performance. Nowadays, the traditional treatment methods are mainly physical cleaning and chemical cleaning, which can improve the surface quality by removing the oxide on the steel surface. Among them, chemical cleaning is easy to use and cheap in the industry [3,4,5], but there are several problems, such as chemical reagent-induced surface damage, low efficiency, environmental pollution, and other problems. Physical cleaning, like mechanical grinding-based cleaning [6], has the disadvantages of low efficiency, high noise, environmental pollution, and other problems, and is unable to satisfy the demands of current green development. Therefore, it is of significant application value to seek economical, green, and environmentally friendly removal processes.

Recently, sand blasting has been one of the most widely used methods in the manufacturing industry. Due to the difference in the degree of rust and sandblasting equipment, the efficiency of rust removal is about 2~6 m^2^/h on the premise of ignoring the process of cleaning residual sand. The cleanliness and the surface roughness of the samples treated by sand blasting can reach Sa 2.5 and 50 μm, respectively [7,8,9]. However, the efficiency of laser cleaning can reach 2.7 m^2^/h, which is close to the efficiency of sand blasting. The cleanliness of the samples treated by laser cleaning can reach Sa 3.0. Moreover, the surface roughness after laser cleaning is not much different from that of the original substrate. By contrast, laser cleaning is considered to be a very promising approach for removing the oxide on the steel surface because it has excellent advantages of high efficiency, non-pollution, and easy automation [10,11,12]. In order to achieve good surface cleaning quality, extensive research work has been carried out on pulsed laser cleaning. Kumar et al. [13] performed pulsed laser cleaning of Ti–3Al–2.5V alloy at repetition rates from 30 kHz to 80 kHz with different fluences and concluded that the threshold fluence at any other repetition rate was found to be between 0.55 J/cm^2^ and 0.80 J/cm^2^ and that laser fluence above 1.75 J/cm^2^ contributed to formation of pits, leading to surface damage. Guo et al. [14] conducted pulsed laser cleaning of SUS409 stainless steel plates and concluded that the highly oxidized surfaces can be cleaned with short, high power density pulses and that the removal of oxide films was mainly attributable to stress wave-induced spallation in the cleaning process. Zhang et al. [15] investigated nanosecond laser cleaning of oxide film on the surface of aluminum alloy and found that the residual oxygen content was closely associated with energy density. Moreover, laser-induced craters altered the surface morphology, subsequently increasing surface roughness. Dong et al. [16] discussed the laser cleaning mechanism for aluminum alloy surface oxide film and affirmed that the removal of surface oxide film was mainly based on the vaporization mechanism and the shock wave induced by the explosion. Daminelli et al. [17] monitored the laser removal process for anodic oxides on an aluminum alloy and proved that nanosecond pulses gave rise to spallation, whereas femtosecond treatment promoted ablation. Li and Guan [18] performed real-time monitoring of laser cleaning for hot-rolled stainless steel and proved that the optimal laser cleaning parameters can avert re-oxidation and decrease the roughness of the cleaned surface. Zhou et al. [19] explored the characteristics of pulsed laser cleaning of aluminum alloys and demonstrated that the oxide laser removal depth and diameter depended mainly on the average power. It was also found that the depth and diameter of the ablation pit increased with the increase in the average laser power during the process of laser cleaning. Garbacz et al. [20] studied the effect of the laser fluence and pulse duration on the microstructure of copper and found that a series of 100-μs pulses provided the most effective cleaning and that the roughness of the cleaned surface was regulated by controlling the number of laser pulses. Wang et al. [21] carried out pulsed laser cleaning of aluminum alloy oxide film and discovered that the removal mechanism of oxide film was mainly based on the gasification mechanism and shock wave induced by explosion. The surface microhardness of aluminum alloy increased from 158.5 HV to 171.9 HV when subjected to laser cleaning. Sun et al. [22] affirmed that the primary mechanism of laser cleaning of rust was the vibration and thermal shock action induced by the pulsed laser, which made rust particles sputter. Psyllaki et al. [23] pointed out that the oxide removal mechanism was mechanical in nature. The stresses produced by laser irradiation on the oxidation layer resulted in the fracture of the interface and the spallation of the surface layer. During the process, the relaxation of the compressive stresses in the thermal oxides greatly heightened their expulsion. Pulsed laser cleaning has significant advantages of small substrate damage and non-pollution during the oxide removal process, but the cleaning efficiency and robust performance still need to be further improved.

In order to address the above problems, continuous laser cleaning has been extensively attempted by many researchers to remove the oxide film on the substrate on account of its combination of high efficiency, robust performance, and affordability. Sun et al. [24] affirmed that the laser cleaning power was positively correlated with the cleaning depth. It was also found that continuous laser irradiation gave rise to heat buildup, and long-term irradiation resulted in the deterioration of surface roughness and performance [25]. Barletta et al. [26] confirmed the suitability of a continuous-wave high-power diode laser to the process of removing oxides from metal surfaces. The research proved that continuous laser cleaning had high efficiency and good roughness in the process of removing oxides. However, oxidation removal did not achieve the expected effect during the continuous laser cleaning process [27,28]. Comparative analysis has found that integrated pulsed-continuous laser cleaning can improve the quality of oxide removal while raising the efficiency. Therefore, it will be a promising method to remove the oxide from substrate surfaces in the manufacturing field.

Current research on oxidation removal is mainly focused on pulsed laser cleaning. However, there are few reports on the study of integrated pulsed-continuous laser cleaning methods. The objective of this work was to investigate the effect of pulsed laser parameters on the quality of surface cleaning for Q235 carbon steel. On this basis, a new cleaning method was innovatively proposed by contrastively studying the oxide removal quality, microstructure, and mechanical properties of experimental samples under different process conditions. Moreover, the oxide removal mechanism for cleaned samples was also explored using integrated pulsed-continuous laser cleaning.

## 2. Materials and Methods

In the experiment, Q235B carbon steel plates (Anshan Iron and Steel Plant, Anshan, China) were used as the sample material. This material is a type of hot-rolled steel. The final rolling and coiling temperatures were 870 °C and 630 °C, respectively. The offered plates were fabricated into experimental samples with dimensions of 10 × 10 × 10 mm^3^ using Model AL400Q cutting machine made by Janpan Laser Sodick. The chemical composition of Q235B carbon steel is listed in Table 1.

**Figure 1:**
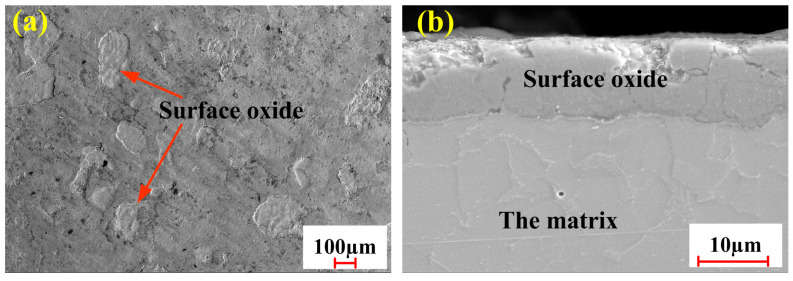
The morphology of the uncleaned sample: (**a**) surface oxide layer; (**b**) cross-section microstructure.

Figure 1 shows the morphology of sample materials in various positions. It can be observed from Figure 1a that the surface of this material is covered by dense oxide films. The thickness of oxide films is up to 12 μm on the substrate surface (Figure 1b). The substrate mainly consisted of ferrite and pearlite structures. The ferrite is a soft structure with good toughness and plasticity, while the pearlite is a hard and brittle structure with high strength and hardness.

**Figure 2:**
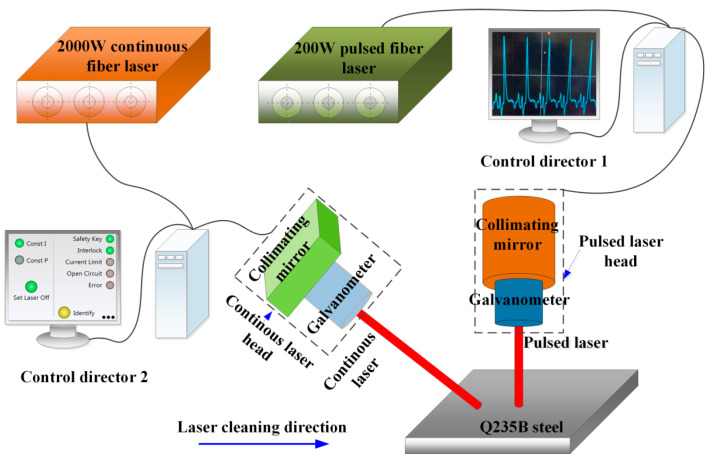
Schematic diagram of experimental setup for integrated laser cleaning.

The experimental lasers in this study were a 2000 W continuous laser and 200 W pulsed laser. The specific parameters of the continuous laser (Model MFSC-2000W laser source made by China laser company Shenzhen Maxphotonics) were as follows: laser power of 10% to 100%, central wavelength of 1080 nm, laser linewidth of 5 mm to 200 mm, and frequency of 0 Hz to 20,000 Hz. The main parameters of the pulsed laser (Model YDFLP-CL-200-1-A laser source made by China laser company Shenzhen JPT Opto-Electronics Co., Ltd., Shenzhen, China) were as follows: average power of 200 W, central wavelength of 1064 nm, laser power range from 10% to 100%, frequency of 5 kHz to 1000 kHz, and scanning width of 5 mm to 150 mm. A schematic diagram of the setup for laser cleaning of Q235B carbon steel plates is shown in Figure 2.

The setting of process parameters was precisely performed by the controller. The cleaning head was tightly installed on the gantry movement mechanism. The gantry movement was precisely controlled through a programmable logic controller (PLC) to obtain the desired speed. The main parameters during the laser cleaning process were laser power, cleaning speed, laser frequency, and scanning width. Process parameters for pulsed laser cleaning are listed in Table 2.

After pulsed laser cleaning, the samples were accurately weighed using an electronic scale (CP224S, Sartorius Stedim Biotech, Göttingen, Germany) to calculate the oxide removal value. A confocal laser scanning microscope (VK-X200K, KEYENCE CO., LTD., Ōsaka shi, Japan) was used for observing the 3D profile of cleaned samples and evaluating physical characteristics. After that, metallographic samples were prepared by mounting, grinding, polishing, and etching processes. In order to reveal the surface morphological evolution of cleaned samples, a scanning electron microscope (SEM, GeminiSEM 300, Carl Zeiss AG, Oberkochen, Germany) was employed to investigate the microstructure at different process parameters. Electron back-scattered diffraction (EBSD, GeminiSEM 300, Carl Zeiss AG, Oberkochen, Germany) was conducted on experimental samples for exploring the grain size, crystal orientation, dislocation density, and grain boundary types. In addition, a nano-indenter was also applied to test the hardness of micro-areas and evaluate the mechanical properties of cleaned samples.

## 3. Results and Discussion

### 3.1. Pulsed Laser Cleaning

#### 3.1.1. Oxide Removal

**Figure 3:**
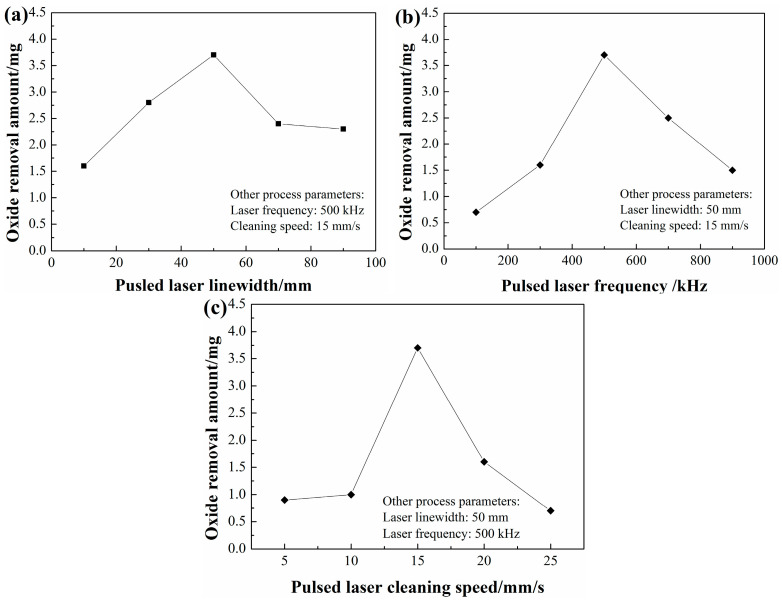
Effect of pulsed laser parameters on oxide removal amount: (**a**) laser linewidth; (**b**) laser frequency; (**c**) laser cleaning speed.

A dense oxide film easily formed on the surface of Q235B carbon steel in the process of hot rolling at high temperature. The presence of oxide film can reduce the absorption rate of the laser during the laser processing process and affect the forming quality of the products. In order to study the effect of pulsed laser parameters on the oxide removal amount, three groups of process experiments were performed on Q235B carbon steel plates. The experimental results for the tested samples are demonstrated in Figure 3.

As shown in Figure 3a, it can be clearly observed that the removal of oxide first increased and then decreased with the increase in pulsed laser linewidth. The maximum value of oxide removal reached 3.7 mg when the pulsed laser linewidth was 50 mm. During the laser cleaning process, the removal of oxide films depended mainly on the laser fluence. Generally speaking, pulsed laser linewidth was inversely proportional to the laser fluence. Although a small pulsed laser linewidth could increase the laser energy during the pulsed laser cleaning process, it also caused a serious remelt on the substrate surface. In such a condition, the removal of oxide films failed to be improved. The remelting phenomenon was gradually suppressed with the increase in pulsed laser linewidth, which efficiently improved the amount of oxide removal. When it exceeded a certain threshold, the oxide began to be removed by the vibration or vaporization induced by the pulsed laser [29]. As the laser linewidth increased persistently, pulsed laser energy could decrease because of the increase in laser irradiation areas, thus resulting in a reduction in oxide removal.

From Figure 3b, it can be found that the oxide removal amount and pulsed laser frequency exhibits a parabolic relationship. The amount of oxide removal reaches its peak at 500 kHz. The total amount of oxide removal during the process of pulsed laser cleaning can be calculated by Formula (1) as follows:

m = ν × m_0_ (1)

where m is the total amount of oxide removal (mg), ν is pulsed laser frequency (kHz), and m_0_ is oxide removal quality under single pulse action (mg). The single pulse energy per second can be calculated by Formula (2) as follows:

E_0_ = P/ν (2)

where E_0_ is the single pulse energy (mJ/s), ν is pulsed laser frequency (kHz) and P is pulsed laser power (W).

When the sample was cleaned at 100 kHz, deep craters were formed because each pulse had a large energy. Unfortunately, the amount of oxide film removal failed to increase during the cleaning process. The main reason was that the energy absorbed by the material was used to increase the depth of craters instead of removing the oxide film. As pulsed laser frequency increased from 100 kHz to 500 kHz, the depth of craters decreased gradually on account of a reduction in single pulse energy. Meanwhile, the amount of oxide film removal increased under the action of single pulse energy. The product of the two reached its maximum value, thus achieving the maximum oxide removal effect. When the sample was cleaned at 900 kHz, the number of pulses reached the maximum. However, the energy of each pulse was too small to remove more oxide films. Therefore, the total amount of oxide film removal would be reduced during the cleaning process.

Meanwhile, the oxide films would be instantaneously punctured and vaporized on account of high energy density. When the laser frequency exceeded a certain threshold, the oxide removal effect was significantly weakened by the reduced energy absorption. As demonstrated in Figure 3c, the amount of oxide removal first increased gradually and then decreased precipitously with increasing cleaning speed. The oxide removal had the maximum value at the cleaning speed of 15 mm/s. The oxide removal amount was closely associated with the energy threshold of the oxide [30]. Although the high average energy was achieved at low pulsed laser cleaning speed, it also led to the remelting of the substrate surface. With an increase in pulsed laser cleaning speed, the remelting was gradually alleviated. Meanwhile, the oxide removal effect was also improved. When the cleaning speed reached a certain threshold, the remelting phenomenon could be fully suppressed. Although the average energy decreased with the increase in the cleaning speed, the oxide could still be removed as long as it exceeded the removal threshold. During the cleaning process, the oxide removal amount would be reduced because of a decrease in average energy. According to the previous analysis, it was concluded that the optimal oxide removal parameters were as follows: pulsed laser linewidth of 50 mm, pulsed frequency of 500 kHz, and cleaning speed of 15 mm/s.

#### 3.1.2. Surface Roughness

In order to investigate the effect of process parameters on physical characteristics, the surface roughness of cleaned samples was carefully examined under different process conditions. The results of surface characterization were used as an index to evaluate the cleaning effect.

**Figure 4:**
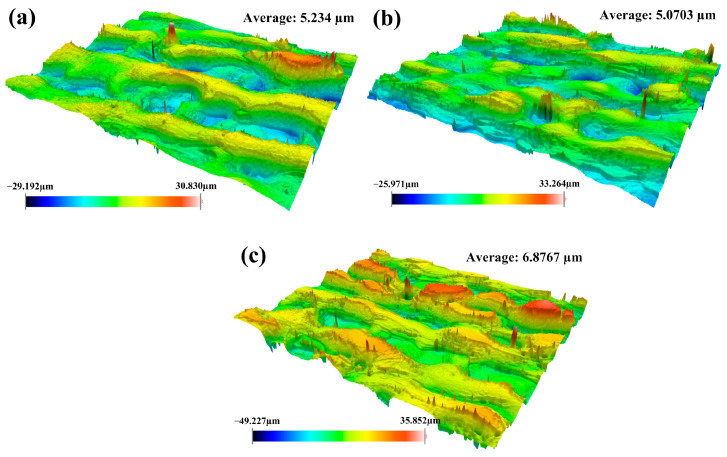
Surface roughness of cleaned specimens with different pulsed laser linewidths: (**a**) 10 mm; (**b**) 50 mm; (**c**) 90 mm.

As demonstrated in Figure 4, it can be clearly observed that the cleaned sample had the smallest roughness at pulsed laser linewidth of 50 mm. It has been also found that the substrate surface developed a great number of craters after laser irradiation. The distribution of these craters showed a staggered pattern. The occurrence of craters during the cleaning process was the result of the interaction between the pulsed laser and the material. The material in the irradiated area could heat up and vaporize rapidly under the action of the pulsed laser with a high peak energy. Thereafter, craters could be easily formed along with the solidification process. Another reason for the formation of craters was the result of the shock of the pulsed laser on the irradiated material. The value of surface roughness was mainly linked to pulsed laser linewidth in the cleaning process. The average laser fluence could increase promptly with the decrease in the laser linewidth, thereby causing in more oxide film to be vaporized and a morphology with large roughness to be formed on the material surface. Moreover, it was also found that the maximum difference in roughness occurred at a laser linewidth of 90 mm. It was mainly determined by laser energy distribution. The larger the laser linewidth, the more dispersed the energy. The uneven energy distribution on the whole surface resulted in a large difference in height.

**Figure 5:**
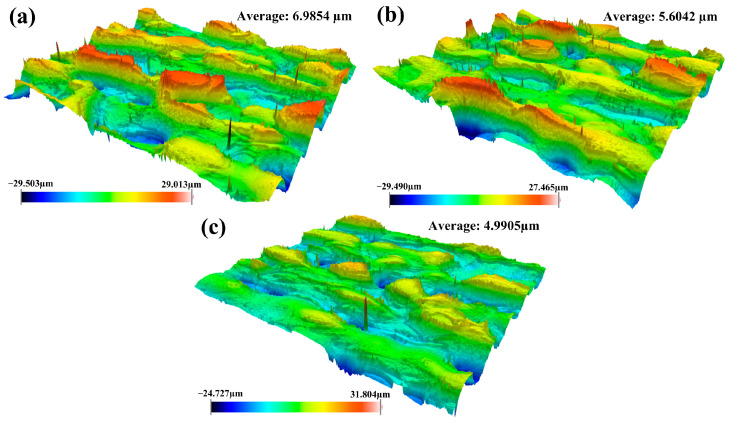
Surface roughness of cleaned specimens with different pulsed laser frequencies: (**a**) 100 kHz; (**b**) 500 kHz; (**c**) 900 kHz.

Figure 5 shows the surface roughness of cleaned samples under different pulsed laser frequencies. It can be found that the surface roughness of cleaned samples decreased with the increase in pulsed laser frequency during the cleaning process. The degree of surface roughness was mainly determined by the depth of the craters. As for the pulsed laser, the smaller pulsed frequency meant the higher single pulse energy. Under this condition, the higher single pulse energy caused more oxides to be removed and formed large craters on the substrate surface. Thus, it can be concluded that it is very important to obtain good surface roughness by selecting the appropriate pulse frequency.

**Figure 6:**
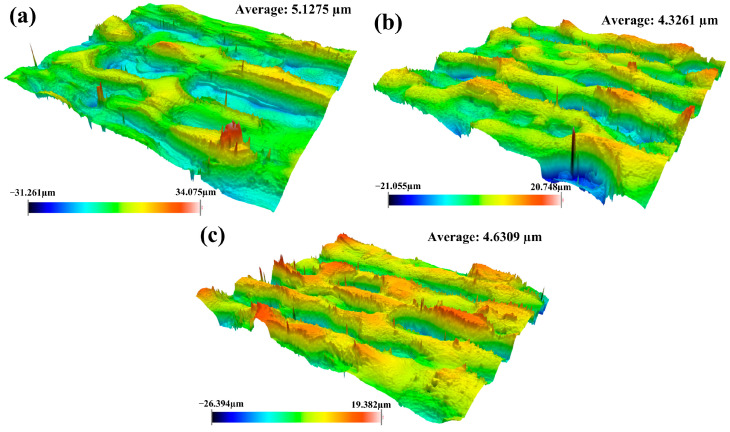
Surface roughness of cleaned specimens with different pulsed cleaning speeds: (**a**) 5 mm/s; (**b**) 15 mm/s; (**c**) 25 mm/s.

As seen from Figure 6, the surface roughness of the material shows a decreasing trend accompanied by the increase in pulsed cleaning speed. During the laser cleaning process, the increase in cleaning speed can decrease the cleaning heat input continually. It can lead to a reduction in oxide films on the substrate surface after laser irradiation. The depth of craters would be further reduced in such a condition. With the decrease in cleaning heat input, the removal pattern of the oxide will be changed.

By analyzing the surface roughness under different process conditions, it was further proved that the suitable pulse cleaning parameters for Q235B carbon steel plates were laser linewidth of 50 mm, pulse frequency of 500 kHz, and cleaning speed of 15 mm/s. Although pulsed laser cleaning technology has obvious advantages in removing the oxide films, it also increases the surface roughness of the material. Therefore, it is necessary to improve the surface performance of materials while removing the oxide films effectively.

### 3.2. Process Optimization

#### 3.2.1. Oxide Removal Under Optimized Process Conditions

**Figure 7:**
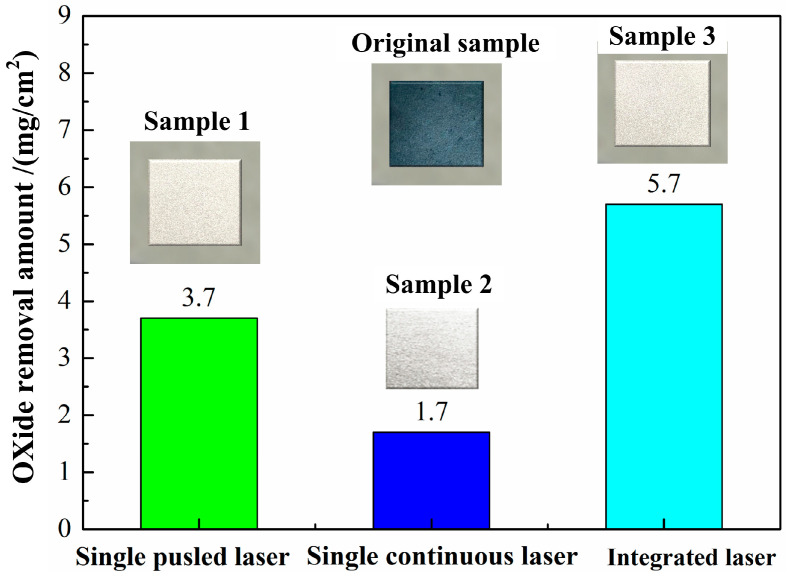
Oxide removal from tested specimens under different cleaning methods.

In order to effectively solve the above problems, different cleaning processes were explored in the following study. It can be clearly observed that the oxide removal amount reached 5.7 mg using integrated pulsed-continuous laser cleaning, apparently higher than that using single continuous laser or pulsed laser cleaning in Figure 7.

The increase in the oxide removal amount may be attributable to increased absorption of laser energy by the materials. After pulsed laser irradiation, many craters can be formed on the substrate surface because of oxide removal. Then, the presence of craters on the surface of the material can fully increase the reflection of the laser when using single continuous laser cleaning. Under this condition, more oxides can be successfully removed.

#### 3.2.2. Surface Roughness Under Optimized Process Conditions

**Figure 8:**
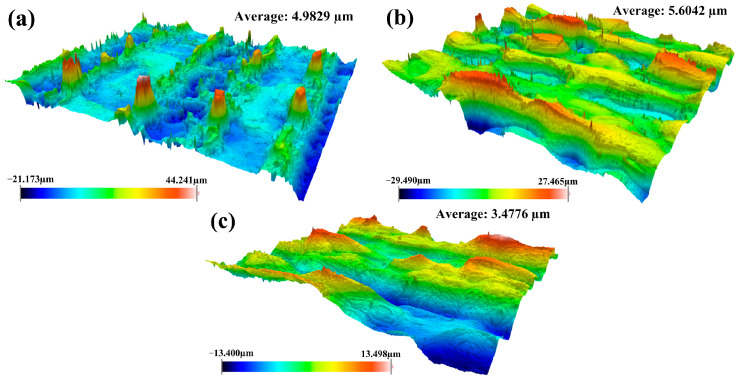
Surface roughness of specimens using different cleaning methods: (**a**) single continuous laser cleaning; (**b**) single pulsed laser cleaning; (**c**) integrated pulsed-continuous laser cleaning.

To further investigate the effect of laser cleaning processes on physical characteristics of the material, the surface roughness of cleaned samples was measured using three cleaning methods. The experimental results for the cleaned samples are demonstrated in Figure 8.

From Figure 8, we observed that the integrated pulsed-continuous laser cleaning had the smallest roughness, followed by continuous laser cleaning and the largest by pulsed laser cleaning. Compared with single pulsed laser cleaning, the improvement in surface roughness for integrated pulsed-continuous laser cleaning was mainly attributed to the remelting effect of continuous laser after pulsed laser cleaning. During the cleaning process, the craters were fully backfilled with excess melted metal around them. The change in morphology led to the improvement in surface roughness.

#### 3.2.3. Micro-Morphology Under Optimized Process Conditions

**Figure 9:**
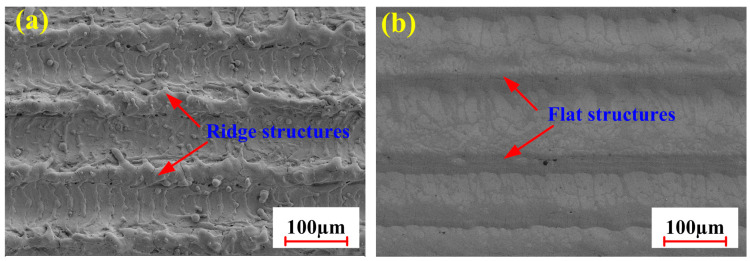
Surface morphology of tested specimens under optimized conditions: (**a**) pulsed laser cleaning; (**b**) integrated pulsed-continuous laser cleaning.

In order to investigate the morphology of tested samples, the SEM results for two samples were analyzed under two cleaning conditions. Figure 9 shows the surface morphology of tested specimens exposed to pulsed laser cleaning and integrated pulsed-continuous laser cleaning.

From Figure 9a, it can be found that the surface of the material formed a furrowed shape after pulsed laser irradiation. The occurrence of typical characteristics depended mainly on the shocking effect of pulsed laser on the material. During the cleaning process, a large number of craters could be formed in the laser irradiation zone. As the heat source moved, these craters were successfully connected to form the trough. Further observation revealed that the wavy morphology was formed in the trough. It was the result of the gasification effect caused by the pulsed laser in the cleaning process.

As demonstrated in Figure 9b, a flat surface could be obtained by integrated pulsed-continuous laser cleaning. It was mainly attributed to the vibration effect of pulsed laser and the remelting effect of continuous laser. Under the effect of pulsed laser irradiation, a large number of craters could be firstly produced in the irradiated area because of instantaneous vaporization of materials. In addition, the oxides in deeper layers became very loose, consequently inducing micro-cracks. With the help of the continuous laser, the loosened oxide films could be easily removed. The peaks also could be melted quickly so as to backfill the troughs.

**Figure 10:**
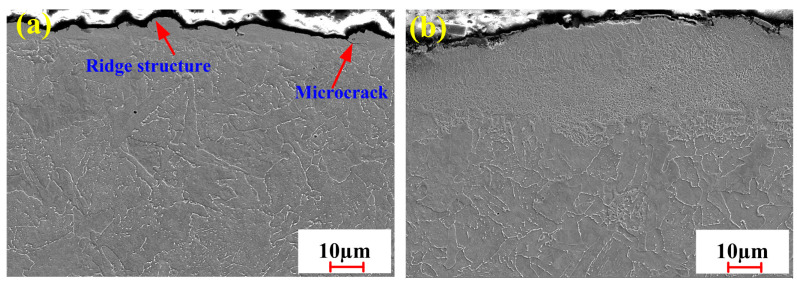
Cross-section morphology of tested specimens under optimized conditions: (**a**) pulsed laser cleaning; (**b**) integrated pulsed-continuous laser cleaning.

The results of closer SEM examination, revealed in Figure 10a, indicated that an irregular and zigzag morphology was formed on the substrate surface. It was mainly determined by the interaction between the material and the pulsed laser. The shocking effect of the pulsed laser on the material accelerated the formation of this morphology. It was also detected that a few cracks occurred between the film layer and the material matrix. The occurrence of the cracks was associated with the thermal stresses produced by the pulsed laser during the cleaning process. During the process of pulsed laser irradiation, the difference in physical properties between the substrate and the oxide resulted in the formation of cracks. It can be observed in Figure 10b that part of the material was removed on the substrate surface. It indicated that the oxide on the substrate surface had been completely removed after integrated pulsed-continuous laser cleaning. Moreover, the surface microstructure of the material obviously could be refined due to the deposition during the cleaning process. This could help to improve the mechanical properties of the material.

#### 3.2.4. Microstructure Under Optimized Process Conditions

To characterize the effect of cleaning processes on the grain size, crystal orientation, dislocation density, and grain boundary types, electron backscatter diffraction tests were performed on selected areas of the samples subjected to pulsed laser cleaning and integrated pulsed-continuous laser cleaning.

**Figure 11:**
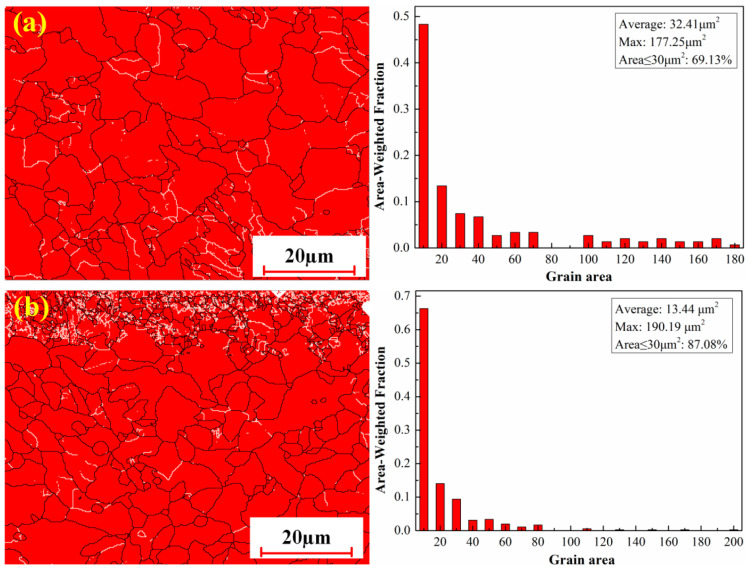
Grain size of cleaned samples with two process conditions: (**a**) pulsed laser cleaning; (**b**) integrated pulsed-continuous laser cleaning.

Figure 11 shows the grain size of cleaned samples with two process conditions. As seen from Figure 11a, the equiaxed structures in the sample exposed to pulsed laser cleaning were relatively uniform. The average grain size was up to 32.41 μm^2^, while the maximum grain area was 177.25 μm^2^ within the selected areas. Whether the grain was transformed depended mainly on the amount of heat input during the cleaning process. Although the pulsed laser had high peak power, the average energy density was very small in the process of radiation due to its own characteristics. For this reason, the grain size near the surface was not significantly affected by the pulsed laser. Additionally, residual oxides on the substrate surface could prevent the transfer of heat, which greatly limited the growth of grains.

Compared with pulsed laser cleaning, the grain area achieved a maximum of 190.19 μm^2^, while the average grain area decreased by 18.97 μm^2^ in integrated pulsed-continuous laser cleaning (Figure 11b). The grains with no more than 30 μm^2^ area increased in size by 25.97% as measured by their cross sectional area. This was mainly due to continuous laser cleaning with relatively large heat input. The increase in maximum grain area was the result of the thermal cycling during continuous laser cleaning. The enrichment of the laser energy resulted in the coarsening of parts of grains. Grain refinement was closely connected with the remelting of surface material in the cleaning process. The remelted metal could solidify rapidly due to the fast cooling speed and then form fine grains. It is very helpful to improve the comprehensive mechanical properties of the material [31].

**Figure 12:**
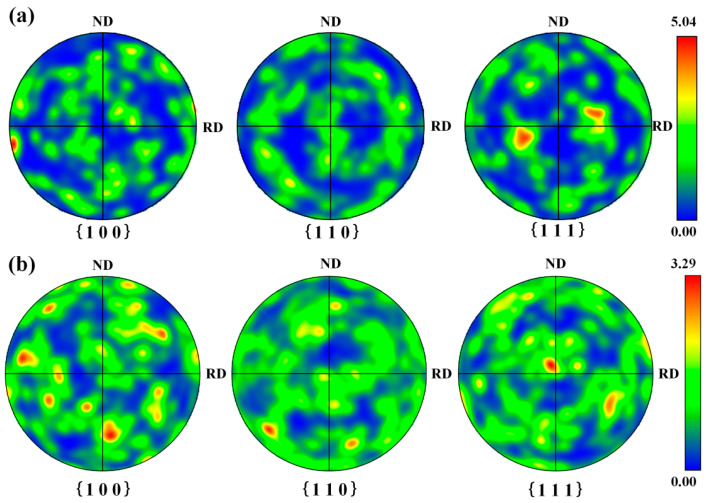
Pole figures of cleaned samples with two process conditions: (**a**) pulsed laser cleaning; (**b**) integrated pulsed-continuous laser cleaning.

Figure 12 demonstrates pole figures of cleaned samples under two process conditions. As shown in Figure 12a, it can be clearly seen that the crystal orientation of Fe-BCC mainly points to the RD direction on the {1 0 0} polar projection plane. The maximum orientation density of the Fe-BCC crystals reaches 5.04. After pulsed laser irradiation, the laser energy was mainly used for oxide removal. Thanks to its small average heat input, the crystal orientation in the substrate was not affected significantly during the cleaning process. In such a condition, the crystal orientation still maintained the original direction (namely, the RD direction).

Compared with pulsed laser cleaning, the trend of the crystal orientation was significantly reduced in the sample subjected to integrated pulsed-continuous laser cleaning in Figure 12b. The maximum orientation density of the Fe-BCC crystals was up to 3.29. It was closely related to remelting of surface metals of the substrate. After continuous laser irradiation, the metal on the surface of the substrate began to melt and solidify quickly. A large degree of supercooling can occur because of fast cooling speed, subsequently resulting in an increase in the nucleation rate. The orientation of the crystals will be restricted on account of the competition between grains during the solidification process.

**Figure 13:**
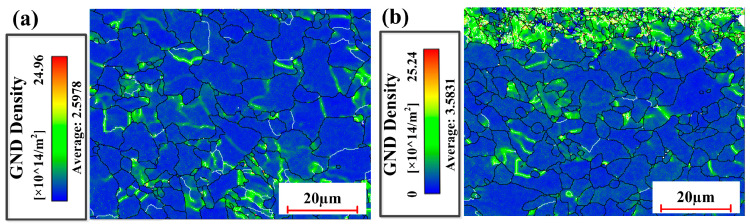
GND density of cleaned samples with two process conditions: (**a**) pulsed laser cleaning; (**b**) integrated pulsed-continuous laser cleaning.

Figure 13 reveals the GND density of the samples under laser cleaning conditions. It can be observed that the average GND density of the sample subjected to pulsed laser cleaning was 2.5978 × 10^12^/m^2^, while that of the sample subjected to integrated pulsed-continuous laser cleaning was 3.5831 × 10^12^/m^2^. The comparison shows that the dislocation density was increased by 37.93%. It depended mainly on the refinement of grains in the sample. The grain refinement can increase the area of grain boundaries. Under the action of external force, the dislocation inside the grain can move towards the grain boundary after starting. The grain boundary can hinder the movement of the dislocation due to large distortion energy at the grain boundary. The dislocation can accumulate at the grain boundary and thus increase the density of the dislocation. The increase in dislocation density is advantageous to the improvement of material strength.

**Figure 14:**
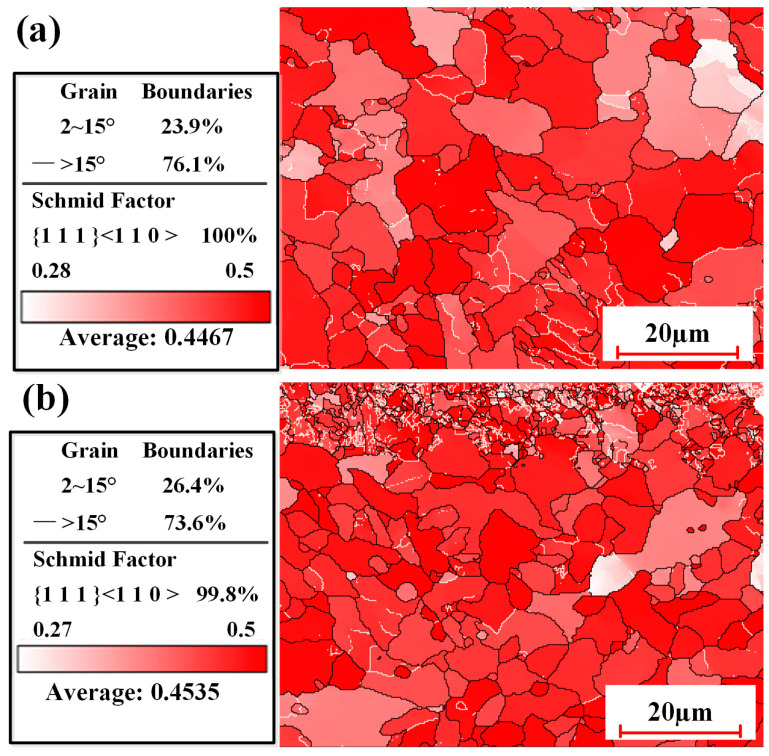
Grain boundaries and Schmid factor of cleaned samples with two process conditions: (**a**) pulsed laser cleaning; (**b**) integrated pulsed-continuous laser cleaning.

Figure 14 demonstrates grain boundaries and the Schmid factor of the samples under two cleaning conditions. It was observed that the proportion of small-angle grain boundaries (2~15°) in the two samples was 23.9% and 26.4%, respectively. Compared with pulsed laser cleaning, the proportion of small angle grain boundaries increased by 10.46% in the sample subjected to integrated pulsed-continuous laser cleaning. The alloy composition is an important factor affecting the small-angle grain boundary. During the solidification process, the composition of the remelt can be altered because of rapid cooling in the air. An increase in small-angle grain boundaries can effectively affect the mechanical properties of the material. Moreover, it was found that the Schmid factor (SF = 0.4535) of the sample with integrated laser cleaning was higher than that (SF = 0.4467) of the sample with continuous laser cleaning. The increase in SF in the sample contributed to the slip of the crystal, effectively improving the plastic deformation ability of the material [32]. Meanwhile, the crack sensitivity was also greatly reduced under the action of external forces.

### 3.3. Micro-Hardness Characterization

**Figure 15:**
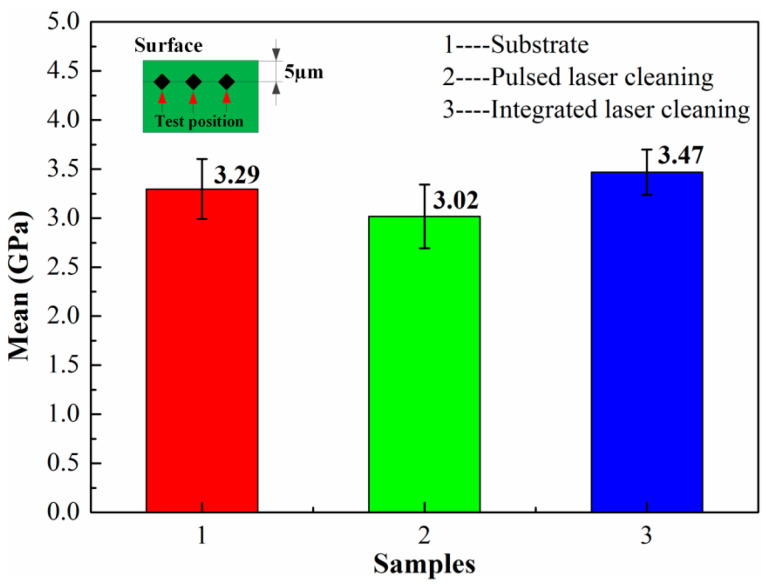
The micro-hardness of samples exposed to different cleaning processes.

To evaluate the mechanical properties of the surface structures of the cleaned samples, the hardness of micro-areas was measured in the samples exposed to pulsed laser cleaning and integrated pulsed-continuous laser cleaning. The main parameters for testing were as follows: 4 mN loading and a load time of 12 s. In order to ensure the reliability of the experimental data, the average values of the three measurements were selected as the test results. Figure 15 graphically depicts the experimental results for the micro-area hardness of cleaned samples subjected to different process conditions.

As shown in Figure 15, the hardness of sample 1 reached 3.29 GPa. It is known from previous studies that it belongs to the oxide rather than the substrate. The high hardness is mainly attributed to its high density. It was also observed that the hardness of sample 2 and sample 3 was 3.02 GPa and 3.47 GPa, respectively. Compared with pulsed laser cleaning, the hardness could be increased by 14.9% in the sample subjected to integrated laser cleaning. The hardness of the material is closely associated with its microstructure. It can be seen from Figure 10 that fine grains were produced near the surface of the sample with integrated laser cleaning. The refinement of grains in the material can greatly increase the area of grain boundaries and hinder the movement of the dislocation. The dislocation must go around or through the grain boundary when the two meet. It can increase the deformation resistance, thereby improving the hardness of the material. Additionally, the refinement of grains can effectively reduce the stress concentration under external forces [33]. In such a condition, plastic deformation of adjacent grains occurs by applying greater external forces on the material.

### 3.4. Oxide Removal Mechanism

**Figure 16:**
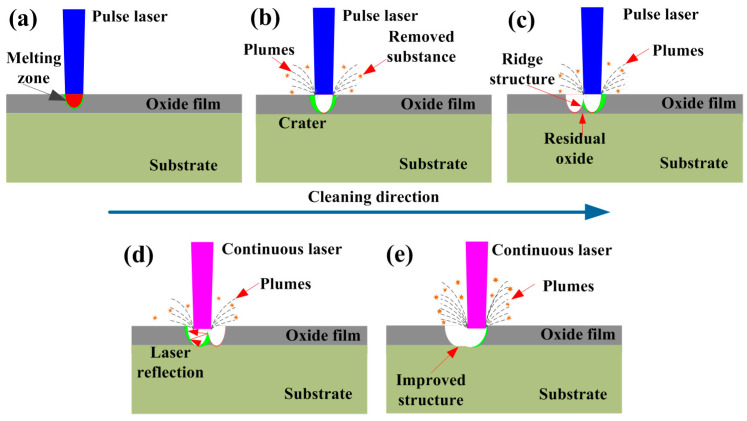
The oxide removal process for Q235B carbon steel subjected to integrated laser cleaning: (**a**) formation of melting zone; (**b**) evaporation and crater generation; (**c**) formation of ridge structures; (**d**) the removal of residue oxide; (**e**) formation of flat structures.

Based on the surface characteristics of Q235B carbon steel under different cleaning processes, a model is proposed to illuminate the mechanism of oxide removal. In Figure 16, we graphically depict the surface oxide removal process for Q235 carbon steel subjected to integrated pulsed-continuous laser cleaning.

After pulsed laser irradiation, the laser energy is rapidly absorbed by the material. The irradiated regions can be firstly melted, subsequently forming a melting zone, as shown in Figure 16a. Although the oxide films are successfully melted in the irradiated regions, they fail to be removed because the temperature is below their boiling point. With the increase in pulsed laser irradiation time, the temperature of the molten pool can rise rapidly and exceed its boiling point. The liquids in the molten pool can quickly vaporize and produce a crater, as shown in Figure 16b. During this process, the plumes can be formed around the crater due to the vaporization and ionization of liquid metal under high pulsed energy. The cleaned products are also overflowed from the molten pool under local overheating conditions.

With the movement of the pulsed heat source, ridge structures can be formed on the cleaned substrate surface, as shown in Figure 16c. It is closely associated with pulsed laser energy distribution during the cleaning process. Most of the oxide film on the substrate surface is removed as expected by the vibration of deep rust deposits and thermal shock action induced by the pulsed laser [34,35,36]. Unfortunately, a small amount of oxide film remains on the substrate surface due to the shortcomings of the pulse itself. After continuous laser irradiation, residual oxides are perfectly removed from the substrate surface, as illustrated in Figure 16d. The existence of ridge structures on the substrate surface will increase the absorption rate of laser energy by repeated reflection when continuous laser irradiates the material. In such a condition, the original craters are further expanded and deepened. Although the energy of the continuous laser could only melt the superficial oxides, the thermal action increased the thermal stress in the deep layer of the oxides, prompting the oxides to detach. Moreover, the increase in the thermal effect in the depth direction could induce the occurrence of micro-deformations at the substrate surface, thus effectively weakening the binding force between the oxide and the substrate. Accompanied by the movement of the continuous laser heat source in Figure 16e, ridge structures on the substrate surface could be successfully eliminated, thus achieving an improved structure. Meanwhile, the micro-deformations induced by the pulsed laser on the substrate surface could be desquamated because of an increase in thermal stresses. Under such conditions, the surface quality of the material could be significantly improved.

## 4. Conclusions

In this work, pulsed laser cleaning was experimentally conducted on Q235B carbon steel. The effects of process parameters on the cleaning quality were carefully investigated by designed single-factor experiments. On this basis, a new cleaning method was innovatively proposed by contrastively studying the oxide removal quality, microstructure, and mechanical properties of cleaned samples under different process conditions. The oxide removal mechanism for the cleaned samples was also graphically demonstrated using integrated pulsed-continuous laser cleaning. The following conclusions were obtained.

(1) Oxide films on the surface of Q235B carbon steel can be successfully removed using pulsed laser cleaning. The optimal process parameters were a laser linewidth of 15 mm, pulsed frequency of 500 kHz, and cleaning speed of 15 mm/s. Under these conditions, the single-pulse energy, the number of laser pulses, and the distribution of the laser spot on the plane reach an optimal balance. The amount of oxide removal reached 3.7 mg with these parameters.

(2) The surfaces of the cleaned samples developed many craters after pulsed laser irradiation. The formation of craters was a result of thermal shock from the pulsed laser on the irradiated material. The expected surface roughness was achieved under optimized operating conditions.

(3) Among three laser cleaning methods, integrated pulsed-continuous laser cleaning had an obvious effect in increasing the oxide removal efficiency and reducing the surface roughness of experimental samples. This was mainly attributable to the increased absorption rate of laser energy and the filling effect of remelted metal induced by continuous laser heating.

(4) The ridge structures could be effectively eliminated from the substrate surface. This is mainly due to the fact that the volcanic crater morphology formed after the action of pulsed laser enhances the absorption of continuous laser energy. Meanwhile, the remelting effect of continuous laser irradiation makes the ridge structure morphology smoother. A fine grain zone was produced on the surface of the substrate because of the high degree of supercooling during the integrated pulsed-continuous laser cleaning process.

(5) Compared with pulsed laser cleaning, the trend of the crystal orientation was significantly reduced in the sample subjected to integrated pulsed-continuous laser cleaning. The average grain area in integrated laser cleaning samples was 13.44 μm^2^, significantly smaller than that of pulsed laser cleaning samples. The grain refinement increased the number of small-angle grain boundaries and the density of the dislocation, which effectively improved the plastic deformation ability of the material itself. Therefore, correspondingly, the average hardness of the integrated laser cleaning samples was about 3.47 GPa, higher than that of the pulsed laser cleaning samples (3.02 GPa).

## Figures and Tables

**Table 1:** Chemical composition of Q235B steel (wt.%).

C	S	P	Si	Mn	Fe
0.20	0.012	0.016	0.12	0.21	Bal.

**Table 2:** Process parameters for pulsed laser cleaning.

No.	Laser Linewidth (mm)	Laser Frequency (kHz)	Cleaning Speed (mm/s)	Oxide Removal Amount (mg)
1	10	500	15	1.6
2	30	500	15	2.8
3	50	500	15	3.7
4	70	500	15	2.4
5	90	500	15	2.3
6	50	100	15	0.7
7	50	300	15	1.6
8	50	700	15	2.5
9	50	900	15	1.5
10	50	500	5	0.9
11	50	500	10	1.0
12	50	500	20	1.6
13	50	500	25	0.7

## Data Availability

The original contributions presented in this study are included in the article. Further inquiries can be directed to the corresponding authors.
